# Prognostic value of patient-reported outcome measures in adult heart-transplant patients: a systematic review

**DOI:** 10.1186/s41687-022-00431-4

**Published:** 2022-03-16

**Authors:** Bernardo Perez Villa, Sultan Alotaibi, Nicolas Brozzi, Kurt P. Spindler, Jose Navia, Jaime Hernandez-Montfort

**Affiliations:** 1 Heart and Vascular Institute, Cleveland Clinic Florida, Weston, FL USA; 2 Heart Center, Segeberger Kliniken GmbH, Bad Segeberg, Germany; 3 Baylor Scott and White Health, Central Texas, USA

**Keywords:** Patient-reported outcomes measures, PROMs, Heart transplantation, Prognostic value, Cost-effectiveness

## Abstract

**Background:**

The aim of this systematic review was to describe the prognostic value of patient-reported outcome measures (PROMs) in adult heart-transplant (HT) patients.

**Methods:**

A systematic search was performed on Ovid Medline, CINAHL Plus, Web of Science, and PubMed. The study protocol was registered on the PROSPERO database (CRD42021225398), and the last search was performed on January 7, 2021. We included studies of adult HT patients where generic and disease-specific PROMs were used as prognostic indicators for survival, readmissions, HT complications, and the onset of new comorbidities. We excluded studies that used clinician-reported and patient-experience outcomes. The Quality in Prognosis Studies tool (QUIPS) was used to measure the risk of bias of the included studies.

**Results:**

We included five observational studies between 1987 and 2015, whose populations’ mean age ranged from 43 to 56 years and presented a higher proportion of males than females. The Kansas City Cardiomyopathy Questionnaire demonstrated a negative correlation with readmissions (coefficient = − 1.177, *p* = 0.031), and the EQ-5D showed a negative correlation with the onset of neuromuscular disease after HT (coefficient = − 0.158, *p* < 0.001). The Millon Behavioral Health Inventory and the Nottingham Health Profile demonstrated a statistically significant association as survival predictors (*p* = 0.002 and *p* < 0.05, respectively). A moderate overall risk of bias was reported in three studies, one study resulted in a low risk of bias, and a proportion of more than 75% of males in each of the studies. High heterogeneity between the studies impeded establishing a link between PROMs and prognostic value.

**Conclusion:**

There is low evidence supporting PROMs usage as prognostic tools in adult HT patients. Comparing outcomes of PROMS to routine prognostic in wider and systematic settings is warranted. Systematic use of PROMs in clinical settings is warranted.

**Supplementary Information:**

The online version contains supplementary material available at 10.1186/s41687-022-00431-4.

## Introduction

As the population ages and the prevalence of heart failure increases, Heart transplantation (HT) has become the definitive lifesaving treatment for end-stage heart failure. HT replaces a diseased heart with a healthy heart donated by deceased donors after brain death or circulatory death with the ultimate goal of increasing patients’ quality of life and lifespan. HT are patients who have end-stage heart failure and HT is the only option when other treatments have failed. End-stage heart failure is mainly secondary to coronary heart disease, although viral infections or hereditary conditions [1].

HT provides the greatest long-term survival for end-stage heart-failure patients [2] and yields improvements in functional status, mental health, and overall quality of life (QoL) [3]. However, the improvement of short- and long-term post-transplant outcomes is jeopardized by the worldwide shortage of donor organs [4]. In the United Kingdom, 174 heart transplants were performed in 2020, which represents a decrease of 5% from the number performed in the previous year [5], while in the same period in Spain, 278 heart transplants were performed, representing 7% decrease compared to 2019 [6]. In the USA, a total of 3,658 HT were performed in 2020, representing an increase of 3% from 2019 [7], although the ratio of transplants performed to available donors decreased [8]. Moreover, there are a limited number of HT centers in the USA, leading to differences in outcomes and quality of care across the country [9]. In addition, the fragility of HT systems was exposed by the COVID-19 pandemic, increasing the total number of waitlist and reducing the HT volume in the USA [10]. Thus, it is of the utmost importance to identify the key risk factors involved in HT and carefully select recipients and donors to optimize patient outcomes while conserving resources.

HT’s challenges are not limited to a shortage of donor organs. After successful HT, patients may suffer the onset of a myriad of new comorbidities such as diabetes, cancer, and opportunistic infections [11–14]. In addition, patients awaiting HT have reported more than 30 preoperative stressors, such as the stigma associated with receiving donated and waiting for heart donors to be found [15].

**Fig. 1:**
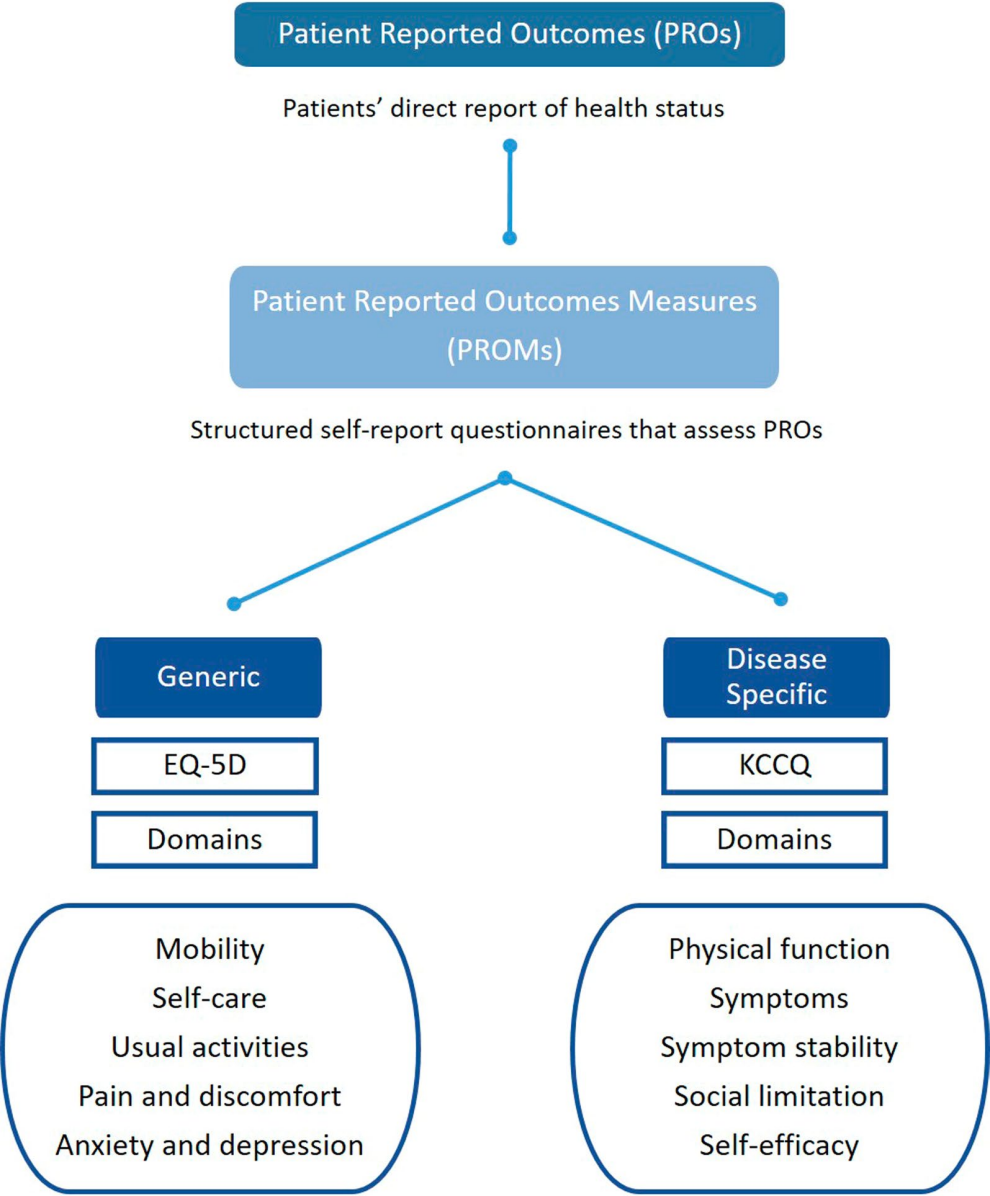
This figure describes PRO's and PROMs' definitions and the two categories of PROMs

In order to measure patients’ health conditions before and after HT, patient-reported outcomes (PROs) provide a direct report of health status from the patients’ perspective while avoiding the interpretive bias of healthcare providers and complement objective health-status measures such as laboratory and imaging tests. The instruments used to assess PROs, usually in the form of structured self-report questionnaires, are denominated patient-reported outcome measures (PROMs) [16], consisting of domains that outline a determined skill or ability. For example, the EQ-5D is one of the most used PROMs in the world, and it is composed of five domains: mobility, self-care, usual activities, pain and discomfort, and anxiety and depression [17]. In addition, PROMs can be designed and validated to assess general aspects of health (generic PROMs) or health-related aspects of a given disease (disease-specific PROMs) (Fig. 1).

Numerous studies have separately studied the impact of HT on quality of life [18] and the prognostic value of PROMs mainly in heart failure populations [19]. However, the independent prognostic value of PROMs in HT patients has not been evaluated despite its promising use to improve decision-making processs [19]. In addition, the use of PROMs in HT patients is not standardized and may be limited due to financial constraints [20]. Thus, a more comprehensive understanding of the actual prognostic function of PROMs in HT patients is imperative to sustain a rationale before incur in the expenditure and efforts of developing a widespread implementation of routine collection of PROMs in HT patients within clinical settings.

We hypothesize that the systematic use of PROMs in HT patients would serve as an independent prognosis tool beyond clinical and laboratory indicators, would equip caregivers with a valid and more comprehensive understanding of the patient’s disease, a better selection and timing of when to perform HT, and in turn improve outcomes, such as mortality, morbidity, and quality of life. Therefore, we conducted a systematic review of the available literature to determine whether PROMs are used systematically in clinical practices and whether they act as prognostic indicators in HT patients.

## Methods

### Search strategy, inclusion and exclusion criteria

We followed the PRISMA group guidelines to identify, select, appraise, and synthesize the studies included in this manuscript [21], and the study protocol was registered on the PROSPERO online database (CRD42021225398) before search execution. We performed a comprehensive search on Ovid Medline, CINAHL Plus, Web of Science, and PubMed, including only studies in English, without a publication date restriction. The last search was performed on January 7, 2021, and the detailed search strategy is presented in Additional file 1: Appendix 1.

Tables 1 and 2 detail the inclusion and exclusion criteria, respectively. In summary, we included studies in adult HT patients where generic and disease-specific PROMs were used as prognostic indicators and collected before, before and after, or after HT, without restrictions regarding the time intervals when PROMS were collected. The intended primary outcomes if data were available were survival and readmissions at 1 month, 6 months, and 1 year, while the secondary outcomes were the proportion of HT complications and the onset of new comorbidities after the HT. We excluded studies where PROMs were not used as prognostic indicators. Studies that used clinician-reported and patient-experience outcomes were also excluded due to the lack of standardization and definition about their use.

**Table 1:** Inclusion criteria

**Population:** heart transplant patients > 18 years old **Intervention:** generic and disease specific instruments developed, validated and tested for measuring patient-reported outcomes **Primary outcomes**: Survival rates at 1 month, 6 months, 1 year Readmissions at 1 month, 6 months, 1 year **Secondary outcomes:** Proportion of heart transplant complications: infections, graft vascular disease, organ rejection, etc Proportion of onset of new comorbidities **Studies:** Randomized clinical trials, observational studies such as case series and case reports involving > = 10 patients, case–control studies, and cohort studies

**Table 2:** Exclusion criteria

**Population:** heart transplant patients < 18 years old **Intervention:** clinician-reported and patient-experience outcomes. Studies where PROMs were not used as prognostic indicators **Studies:** case series and case reports involving < 10 patients, narrative, scope, systematic reviews or meta-analysis, editorials, education papers, conference abstracts, protocols, guidelines, reports, theses, or book chapters	

After duplicates were removed, two independents reviewers (BPV and SA) screened the abstracts by the titles resulted from the research. The full-text analysis followed, based on the inclusion and exclusion criteria and independently completed by two reviewers (BPV and JHM). A third independent reviewer (SA) resolved any disagreements at this stage. Two independents reviewers (BPV and JHM) manually extracted data regarding population and study characteristics, as well as the PROMs’ domains and study outcomes.

### Risk of bias assessment

The Quality In Prognosis Studies (QUIPS) tool was used to measure the risk of bias of the included studies. QUIPS evaluates the validity and the biases in studies of prognostic factors [22]. Two independent reviewers (BPV and JHM) performed the risk of bias assessment, and a third independent reviewer (SA) resolved any disagreements, if necessary.

### Data analysis and synthesis

We summarized the characteristics of the included studies (study design, follow-ups, and mean age) and the characteristics of the PROMs used in the studies (type of PROMs, the statistical model used to determine the prognostic value, and the measures of effect, such as regression coefficient values or hazard ratios). However, we were unable to perform a meta-analysis, because the included studies used different PROMs, measured different outcomes, and applied different follow-up intervals, resulting in high heterogeneity between the studies and impeding their comparison.

## Results

### Eligible studies and overview of study characteristics

**Fig. 2:**
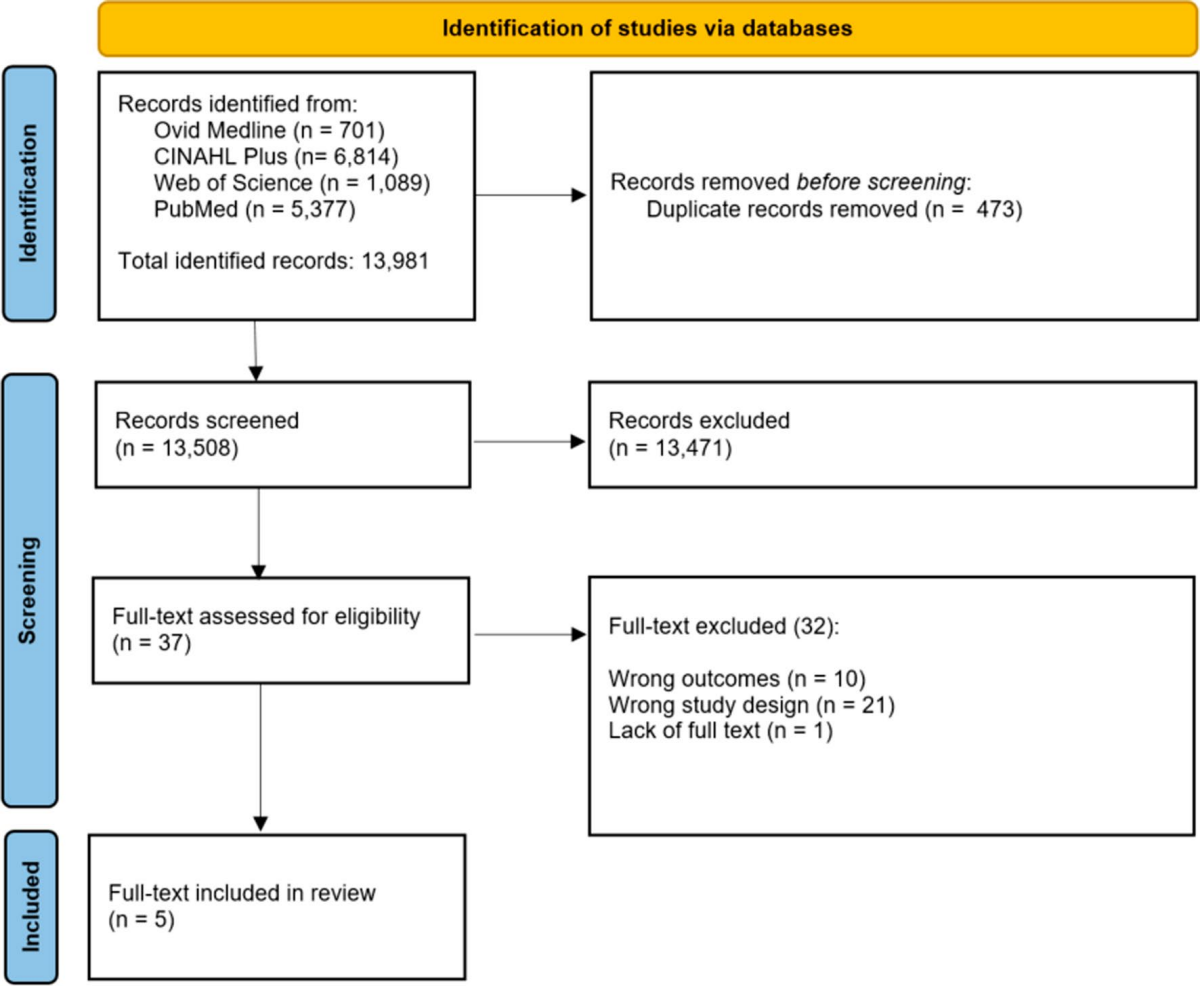
.

A total of 13,981 records were identified from systematic searches in four databases (Ovid Medline, CINAHL Plus, Web of Science, and PubMed). After 473 duplicated references were eliminated, we screened 13,508 titles and abstracts, assessing 37 full-text studies for eligibility (see Fig. 2). The main reason for excluding studies was a wrong study design, which were studies that did not meet the inclusion criteria based on the population of study, the use of generic and disease-specific PROMs as prognostic tools, outcomes, and type of study (see Table 1). A detailed list of the excluded studies with rationales is available in Additional file 1: Appendix 1.

We included five observational studies between 1987 and 2015, whose populations’ mean age ranged from 43 to 56 years, and a proportion of more than 75% of males in each of the studies (Table 3). We did not identify randomized clinical trials that evaluated PROMs as prognostic indicators after our systematic review. PROMs were collected before and after HT in three studies [23–25] and only after HT in two studies [26, 27], with follow-up intervals that ranged from three months to 10 years after HT. Three studies were from the United States, one was from Spain, and one was from the United Kingdom.

**Table 3:** Characteristics of the included studies

Author	Study design	PROM measurement	Period of enrollment	Mean age (SD)	Gender	Participation in the study by eligible individuals	Proportion of patients that completed PROM	Country
Delgado, 2015	Multicenter Observational Prospective	At 6, 12, 36, 60, and 120 months after HT	December 2010 and December 2011	56.4 (11.4)	77.9% male 22.1% female	331 of 350 (94.57%)	86.6% (303 of 350 HT patients)	Spain
Farmer, 2013*	Multicenter Observational Prospective	At enrollment before HT and every 6 months during follow-up. The average follow-up per patient was 2.5 years	July 1, 1990 to June 30, 1999	54 (–)	79% male 21% female	597 of 885 (67%)	93.0% (555 of 597 HT patients)	USA
Harper, 1998	Observational Prospective	At enrollment before HT	From November 1989 to June 1994	53.16 (11.19)	86% male 14% female	90 of 136 (66%)	100% (90 of 90 HT patients)	USA
O’Brien, 1987	Observational Prospective at 2 medical centers	Before HT, once patients where accepted for HT at 3 months intervals After HT at 3 months intervals during 4.5 years	From April 1982 to 30 June 1985	42.8 (–)	92.8% male 7.2% female	73 of 221 (33%)	100% (73 of 73 HT patients)	UK
White-Williams, 2013*	Retrospective analysis of a prospective longitudinal study at 4 medical centers*	At 5 and 10 years after HT	July 1, 1990 to June 30, 1999	53.8 (–)	78% male 22% female	597 of 884 (68%)	39% (216 of 555 HT patients)	USA

*Same cohort of HT patients

The proportion of patients that participated in the study by eligible individuals ranged from 33 to 94%, while the proportion of patients that completed PROMs was higher than 85% in all the studies except for White-Williams et al.’s which reported 39% completion of PROMs.

### Qualitative assessment

Table 4 depicts the QUIPS risk-of-bias assessment for the five included studies, including an overall risk assessment based on six domains: study participation, study attrition, prognostic-factor measurement, outcome measurement, study confounding, statistical analysis-reporting. A moderate overall risk of bias was reported in three studies [23, 25, 26], one study resulted in a low risk of bias [27] and one resulted study in a high risk of bias [24]. The detailed risk-of-bias assessment for each of the included studies is available in Additional file 2: Appendix 2.

**Table 4:** Risk of bias assessment

Author	Study participation	Study attrition	Prognostic factor measurement	Outcome measurement	Study confounding	Statistical Analysis and reporting	Overall risk of bias
Delgado (2015)	Low	Moderate	Low	Moderate	Moderate	Low	Moderate
Farmer (2013)	Moderate	Moderate	Moderate	Moderate	Moderate	Low	Moderate
Harper (1998)	Moderate	High	High	Moderate	High	Moderate	High
O’Brien (1987)	Moderate	High	Moderate	Moderate	Moderate	Low	Moderate
White-Williams (2013)	Moderate	Moderate	Moderate	Low	Low	Low	Low

### Descriptive analysis: PROMs as a prognostic indicator in HT

All the studies performed multivariable prognostic-model analysis to correlate PROMs and outcomes. PROMs demonstrated a statistically significant correlation as a prognostic indicator in four of the five included studies [23–26] (see Table 5). Generic and disease-specific PROMs were used in three studies [23, 26, 27], while the remaining two studies used only generic PROMs [24, 25] (see Table 6).

**Table 5:** PROMs as prognosis indicator

Author	Statistical model	PROMs	Prognosis indicator	
Delgado, 2015	Multivariate regression analyses	EQ-5D	Neuromuscular disease: coefficient value − 0.158 (− 0.240 to − 0.075, *p* < 0.001) Urological disease: coefficient value − 0.183 (− 0.301 to − 0.066, *p* < 0.001)	
		KCCQ overall score	Readmissions: coefficient value − 1.177 (− 2.243 to − 0.112, *p* = 0.031) Graft vascular disease: coefficient value − 10.198 (− 18.219 to − 2.178, *p* = 0.013)	
Farmer, 2013	Multivariate regression analyses	Quality of Life Index Satisfaction Social and economic satisfaction domain	Mortality at 5 to 10 years after HT: Hazard Ratio 0.05 (0.00–0.75), *p* = 0.03	
Harper, 1998	Multivariate regression analyses	Millon Behavioral Health Inventory	**Prediction of survival:** MBHI scale between 17 of the 20, indicating high stress and difficulties coping, was a predictor of survival, *p* = 0.002* Prediction of Post-transplant care required** Pain Threat Responsivity, coefficient value 0.44*, *p* = < 0.001 Cooperative coping style, coefficient value 0.21*, *p* = 0.037 **Post-transplant Infection Rate** Future Despair, coefficient value 0.65*, *p* = 0.001 Life Threat Reactivity, coefficient value − 0.44*, *p* = 0.02	
O’Brien, 1987	Multivariate regression analyses	Nottingham Health Profile	Percentage of all NHP pre-transplant score affirmed	Relative mortality risk
			0	1.00
			20	2.07
			40	4.29
			60	8.89
			80	18.41
			100	38.11
White-Williams, 2013	Multivariate regression analyses		None of the PROM’ domains showed statistically significant prognostic value for survival or other outcomes	

* Confidence intervals not informed

** Post-transplant care was evaluated by a care rate index defined as the hospitalization plus outpatient visit days over the number of days of survival

**Table 6:** Details of PROMs and PROMs’ domains used in the included studies

Study	PROM	Generic or disease specific	Domains		Comments
Delgado, 2015	EQ-5D-3L	Generic	Mobility Self-care Usual activities Pain/discomfort Anxiety/depression		A visual analog scale that ranges from 0 (worst state) to 100 (perfect health) is used to determine patients’ perceived Health Related Quality of life
	Kansas City Cardiomyopathy Questionnaire (KCCQ)	Disease specific	Physical Function Symptoms Symptom stability Social limitation Self-efficacy Quality of life		Two summary scores can be calculated: Overall summary score (OSS): includes the total symptom, physical function, social limitations and quality of life scores Clinical summary score (CSS): includes total symptom and physical function scores to correspond with NYHA Classification The scores are converted into a scale ranging from 0 (worst level) to 100 (highest level)
	Duke-UNC Functional Social Support Questionnaire	Generic	This eight-item PROM measures the strength of the person's social support network		Scoring ranges from 11 (lowest level of social support) to 55 (highest level) A score of ≥ 32 indicates a normal social support network
	Zarit Caregiver Burden Interview	Generic	This 22-item PROM measures the impact of the patient’s condition on caregivers’ life, stress, and burden		The closest relative or caregiver of each patient completed this PROM Scoring ranges from 0 (lowest burden) to 88 (highest burden)
Farmer, 2013 and White-Williams, 2013	Ferrans and Powers Quality of Life Index	Generic	Health and functioning Psychological/spiritual domain Social and economic domain Family		
	Heart Transplant Symptom Checklist	Disease specific	The Heart Transplant Symptom Scale measures 92 symptoms related to heart disease and heart failure, transplantation, medication side effects, and complications commonly found in this population		
	Heart Transplant Stressor Scale	Disease specific	Finding out about the need for a transplant Having end-stage heart disease Family worrying Illness symptoms Waiting for a donor	Uncertainty about the future No energy for leisure activities Constantly feeling worn out Less control over life Dependency on others	
	Sickness Impact Profile	Generic	Sleep and rest Eating Work Home management Recreation and pastimes Ambulation	Mobility Body care and movement Social interaction Alertness behavior Emotional behavior Communication	The overall maximum score is 100% 0% represents a good health status without physical or behavioral changes due to illness 100% represents a poor health status or a major impact of illness on behaviour
	Jalowiec Coping Scale	Generic	Confrontive (10 items) Evasive (13 items) Optimistic (9 items) Fatalistic (4 items) Emotive (5 items) Palliative (7 items) Supportive (5 items) Self-reliant (7)		Consists of 60 coping behaviors
	Heart transplant Social Support Index	Disease specific	5 questions addressing emotional support 10 questions addressing tangible support		This PROM measures the structural aspects of the social support network, and satisfaction with support (emotional, tangible, and overall)
	Positive and Negative Affect Schedule	Generic	Fear Sadness Guilt Hostility Shyness	Fatigue Surprise Joviality Self-Assurance Attentiveness Serenity	
Harper, 1998	The Millon Behavioral Health Inventory	Generic	8 coping/adjustment styles associated with personality types 6 psychogenic attitude scales 3 psychosomatic correlates scales 3 prognostic indices scales		Consists of 60 coping behaviors
O’Brien, 1987	Nottingham Health profile	Generic	6 dimensions of social functioning: Energy Pain Emotional reactions Sleep Social isolation Physical mobility	7 life areas affected Work Looking after the home Social life Home life Sex life Interests and hobbies Vacations	

Delgado et al. showed a statistically significant negative correlation between the generic PROMs EQ-5D and post-HT neuromuscular and urological diseases (coefficient = − 0.158, *p* < 0.001, and coefficient = − 0.183, *p* < 0.001, respectively), while the disease-specific Kansas City Cardiomyopathy Questionnaire (KCCQ) showed a statistically significant negative correlation with readmissions (coefficient = − 1.177, *p* = 0.031) and vascular-graft diseases (coefficient = − 10.198, *p* = 0.013).

Farmer et al.’s and White-Williams et al.’s studies were part of the same prospective cohort that analyzed the health-related quality of life outcomes in HT patients using three disease-specific PROMs and two generics PROMs (see Table 6). Farmer et al. showed that the social- and economic-satisfaction domain of the Quality of Life Satisfaction Index presented a hazard ratio of 0.05 for mortality at five and 10 years after HT ( 95% CI [0.00, 0.75], *p* = 0.03). However, this correlation was not demonstrated by White-Williams et al.

O'Brien et al. described the Nottingham Health Profile (NHP) prognostic value score, a generic PROM with a scale between zero and 100 where a lower scorer represents a higher quality of life. The NHP showed a positive correlation with mortality (coefficient = 0.0364, *p* < 0.05).

Harper et al. evaluated the generic PROM Millon Behavioral Health Inventory (MBHI), which was a statistically significant predictor of survival (*p* = 0.002). The MBHI domains future-despair domain and life-threat reactivity were associated with higher post-transplant infection rate (coefficient = 0.65*, *p* = 0.001, and coefficient = − 0.44*, *p* = 0.02, respectively). The authors also estimated the utility of the MHBI in predicting post-transplant care using the care-rate index, which reflected the use of post-HT care by weighting the days of hospitalization, the outpatient visit days, and the number of days of survival. The care-rate index showed a statistically significant positive correlation with pain-threat responsivity and cooperative-coping style (coefficient = 0.44, *p* < 0.001, and coefficient = 0.21, *p* = 0.037, respectively).

## Discussion

To our knowledge, this is the first comprehensive systematic review that evaluated PROMs as prognostic indicators in HT patients. We reported that PROMs demonstrated statistically significant prognostic prediction in four of the five included studies [23–26]. Farmer et al. and White-Williams et al. studied the same cohort of patients as part of a prospective study of health-related quality of life. While Farmer et al. described a statistically significant association between PROMs and survival, this association was not observed by White-Williams et al. The remaining three studies that showed statistically significant prognostic value presented high heterogeneity, which impeded a comparison between studies. Therefore, the results of this systematic review are limited to support the value of PROMs as a prognostic indicator in HT patients.

Previous systematic reviews have evaluated the value of PROMs as prognostic indicators, especially in cancer populations for overall and disease-free survival [28]. Moss et al. identified several domains of QoL as potential prognostic indicators for oncological outcomes in tumors of the pelvic abdominal cavity [28]. Unlike Moss et al.’s study, our systematic review lacks the rigorous evidence to make these assumptions and we are unable to extrapolate these results to heart transplant patients. However, these strong conclusions should be weighted due to the high heterogeneity that Moss et al.’s faced in the design and methodology of their included studies, which impeded the performance of a meta-analysis, as in our study.

Regarding studies similar to ours in the cardiovascular field, Kelkar et al.’s systematic review was focused on disease-specific PROM in heart failure in clinical care, identifying only two out of nine PROMs with prognosis value: the KCCQ and Minnesota Living with Heart Failure Questionnaire (MLHFQ) [19]. Indeed, the KCCQ and the MLHFQ are two disease-specific PROMs that have been demonstrated as prognostic indicators of heart-failure readmissions and deaths [30–32]. Moreover, PROMs may have more prognostic value than classifications used in daily clinical practice in heart failure patients. Greene et al. recently demonstrated a greater than 50% discordance between the New York Heart Association (NYHA) class and the KCCQ overall score in heart-failure patients. The authors demonstrated that changes in the KCCQ overall score might have a better prognostic value than the NYHA class, stating that an improvement of five or more points in the KCCQ overall score was independently associated with decreased mortality (hazard ratio = 0.59, 95% CI [0.44, 0.80], *p* < 0.001) [30]. Despite the temptation to generalize these results to heart transplant patients, our study suggests that the existing disease-specific PROMs in HT are insufficient to be used with prognostic value.

Why PROMs are not widely implemented in patients undergoing HT?

There is a lack of routine and structured use of PROMs in patients undergoing HT, despite the fact they are recommended for systematic use in cardiovascular diseases [31] and their use as endpoints for trials has grown in the last decade [19, 32]. A systematic review of the role of PROMs in contemporary randomized controlled trials in cardiology showed that PROMs were used in 16% of randomized controlled trials published in major cardiology and general-medical journals between 2005 and 2008 [32]. The limited use of PROMs in specific cardiovascular diseases was highlighted by Chen et al., showing that only 18% (43 of 237) of randomized clinical trials reported using PROMs used PROMs in cardiac catheter ablation for treating symptomatic arrhythmias [33].

A potential explanation for the limited use of PROMs could be the potential delays and disruptions of clinical workflows produced by them, as well as the lack of infrastructure and integration in clinical workflows. However, PROMs have been established as a standard of care in HT clinical practices after demonstrating a reduction in the duration of medical visits without affecting the quality of care and being accepted by patients and healthcare providers [29, 34].

Another concern that may have impeded the routine implementation of PROMs is their lack of capacity to weigh the influence of financial resources, education, and the burden on family caregivers on outcomes. PROMs should systematically capture socioeconomic factors, health care access, and different practice patterns that may have associations with disease outcomes while facilitating informed clinical decision-making. For example, in cardiovascular trials PROMs were found to be crucial for informed clinical decision making in almost a quarter of 413 trials but their role was uncertain in a fifth and irrelevant in 5% of them [32].

Finally, the development and deployment of PROMs in clinical settings may be constrained by the costs of hiring additional personnel, the acquisition of new equipment, and follow-up costs.

What characteristics should PROMs have to be a standard of care in HT patients?

**Fig. 3:**
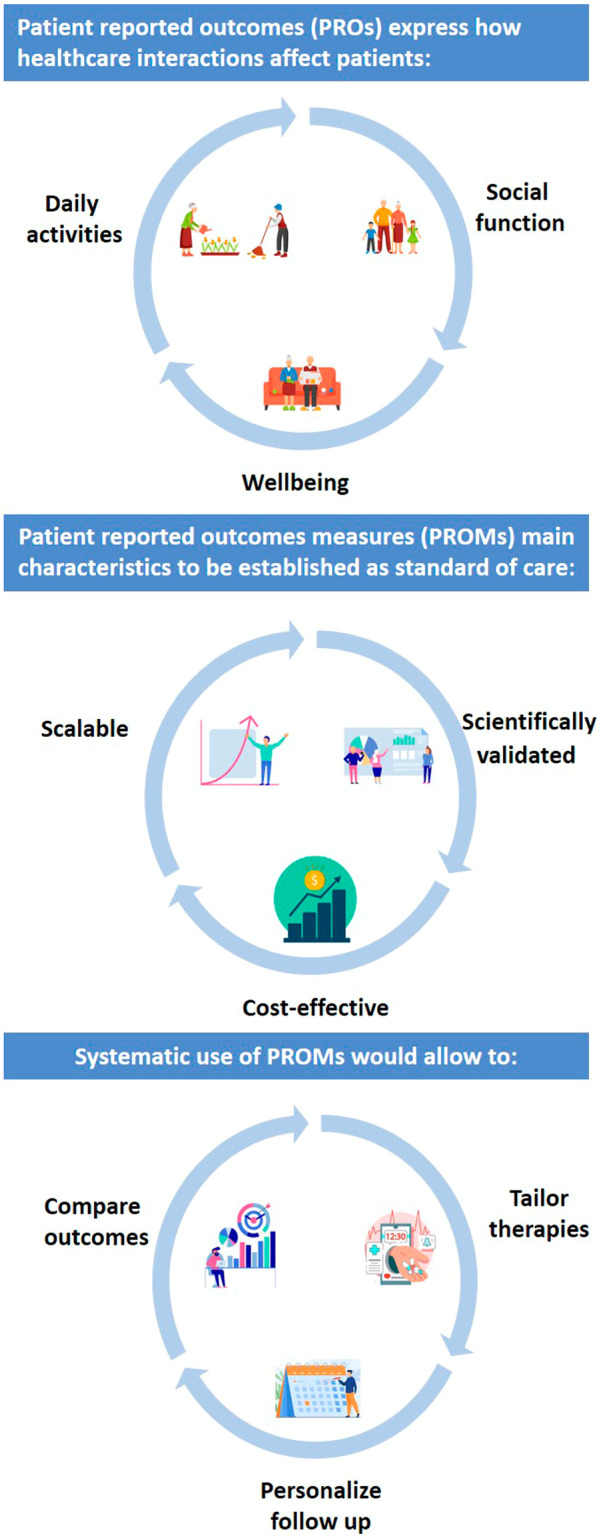
This figure depicts PROMs' main characteristics to be established as a standard of care and the benefit of their utilization

In order to be established as a standard of care in clinical settings where HT are performed, we propose that PROMs meet three characteristics: cost-effectiveness, scientific validation, and scalability (see Fig. 3). PROMs that meet these characteristics have captured > 95% of baseline PROs, > 95% of disease severity and treatment outcomes, and > 70% of one-year follow-ups across multiple clinical settings [35].

PROMs expand beyond traditional functional status measures and longevity improvement, assessing a broader impact on patients’ health status and potentially influencing clinical decision-making. Cost-effective PROMs should allow data capture using existing software and hardware infrastructure while migrating from manual to automated follow-up methods[36]. In order to ensure scalability in tight-scheduled and resource-constrained clinical practices, PROMs should be easy to administer, allow automated data collection and scoring of the assessed clinical event, and provide understandable results for patients and caregivers.

In order to limit unintended consequences, PROMs have to be scientifically validated and supported by peer-review publications, demonstrating content and construct validity [19]. Content validity warrants that PROMs consider specific outcomes for a specific population and the participation of a specific population in the development of the instrument [19]. Complementary PROMs with construct validity demonstrate correlations and detect changes over time between PROMs’ results and clinically meaningful measures [19]. For example, the disease-specific KCCQ for heart-failure patients demonstrated content validity by involving them in its development, while it demonstrated construct validity by correlating its results with the six-minute walk test and peak-exercise oxygen consumption [37].

By achieving cost-effectiveness, scientific validation, and scalability, PROMs would consolidate as a cross-sectional and longitudinal standard of care to improve clinical decision-making by, for example, modifying follow-up intervals, involving other providers, comparing outcomes after treatments, individualizing risk stratification, and providing tailored treatment (see Fig. 3).

Additional practical considerations to facilitate the implementation of PROMs are the ease of data collection, the level of collaboration among colleagues, the provision of clear guidelines for implementation and data collection, the level of managerial involvement, the availability of training and support, and the use of technology [38]. Finally, from a provider perspective, PROMs should allow to interpretability and validity of the information [38].

### Limitations

The major limitation of our study is the inclusion of a small number of observational studies between 1987 and 2015 with PROMs being collected at very different time intervals, time points, and comparing multiple types of outcomes. Observational studies lack sound methodological guidance compared to randomized clinical trials, which reduces the strength of potential recommendations and conclusions made in our study [39]. Most observational studies present different designs, complicating data extraction and outcomes comparison, increasing the probability of errors and affecting the synthesis of evidence. The difference in study designs, collection of PROMs at different time points, and outcomes were evident in our systematic review, where three studies strategized the collection of PROMs before and after HT [23–25] and two studies only after HT [26, 27]. In addition, the included studies in our systematic review were held in three different countries (the United States, Spain, and the United Kingdom), which limited the comparison of results among the included studies due to the different delivery, infrastructure and organization of healthcare systems among countries.

Another limitation of observational studies is the complexity of the risk of bias assessment tools compared to the tools used for randomized clinical trials. We used QUIPS, a risk of bias tool validated for prognostic factors [22], which requires assessing six domains (study participation, study attrition, prognostic-factor measurement, outcome measurement, study confounding, and statistical analysis-reporting). The overall risk of bias was moderate to high in four of the five included studies, indicating a high heterogeneity between studies due to different PROMs used to evaluate the prognostic value, different follow-up intervals, and lack of definition of new comorbidities after the HT. Thus, we did not perform a quantitative comparative analysis between the included studies and therefore the external validity of our results is limited.

However these limitations, our study has shed light on the need to conduct randomized clinical trials where generic and disease-specific PROMs are systematically used to compare their prognostic value with usual care. Such research will elucidate PROMs’ prognostic value and lead to their incorporation as permanent tools to aid clinical decision-making.

## Conclusion

We reported limited evidence supporting the use of PROMs as a prognostic indicator in adult HT patients. Scalable PROMs should be implemented as an integral part of rigorous clinical research settings to further assess their usefulness, enable their optimization for routine clinical practice, and develop of heart transplant specific PROMs that support person-centered care.

## Supplementary Information

**Additional file 1**. Appendix (PRISMA checklists, search strategy, excluded studies with reasons).

**Additional file 2**. QUIPS Risk of bias assessment instrument for prognostic factors studies.

## Data Availability

Data sharing is not applicable to this article as no datasets were generated or analyzed during the current study.

## Abbreviations

HT: Heart transplantation
KCCQ: Kansas City Cardiomyopathy Questionnaire
MBHI: Millon Behavioral Health Inventory
MLHFQ: Minnesota Living with Heart Failure Questionnaire
NHP: Nottingham Health Profile
PROs: Patient-reported outcomes
PROMs: Patient-reported outcome measures
QoL: Quality of Life
QUIPS: Quality In Prognosis Studies
