# Supplementary material for: Prognostic value of patient-reported outcome measures in adult heart-transplant patients: a systematic review
Source: J Patient Rep Outcomes. 2022 Mar 16;6:23. doi: 10.1186/s41687-022-00431-4 (PMC8924738; doi:10.1186/s41687-022-00431-4)
Supplement: Supplementary file 1 — Additional file 1. Appendix (PRISMA checklists, search strategy, excluded studies with reasons). [file 41687_2022_431_MOESM1_ESM.docx]

# Appendix

## PRISMA checklist

| **Section and Topic** | **Item #** | **Checklist item** | **Location where item is reported** |
| --- | --- | --- | --- |
| **TITLE** | | |  |
| Title | 1 | Identify the report as a systematic review. | Title page |
| **ABSTRACT** | | |  |
| Abstract | 2 | See the PRISMA 2020 for Abstracts checklist. | See section PRISMA 2020 for abstract checklist |
| **INTRODUCTION** | | |  |
| Rationale | 3 | Describe the rationale for the review in the context of existing knowledge. | Page 1 paragraphs 1, 2 and 3 |
| Objectives | 4 | Provide an explicit statement of the objective(s) or question(s) the review addresses. | Page 1 paragraph 3 |
| **METHODS** | | |  |
| Eligibility criteria | 5 | Specify the inclusion and exclusion criteria for the review and how studies were grouped for the syntheses. | Tables 1 and 2 |
| Information sources | 6 | Specify all databases, registers, websites, organisations, reference lists and other sources searched or consulted to identify studies. Specify the date when each source was last searched or consulted. | Page 2, section **Methods**, subsection **Search strategy, inclusion and exclusion criteria** |
| Search strategy | 7 | Present the full search strategies for all databases, registers and websites, including any filters and limits used. | See below section **Search strategy and number of studies found** |
| Selection process | 8 | Specify the methods used to decide whether a study met the inclusion criteria of the review, including how many reviewers screened each record and each report retrieved, whether they worked independently, and if applicable, details of automation tools used in the process. | Page 2 section **Methods** Subsection **Study selection and data extraction** |
| Data collection process | 9 | Specify the methods used to collect data from reports, including how many reviewers collected data from each report, whether they worked independently, any processes for obtaining or confirming data from study investigators, and if applicable, details of automation tools used in the process. | Page 2 section **Methods** Subsection **Study selection and data extraction** |
| Data items | 10a | List and define all outcomes for which data were sought. Specify whether all results that were compatible with each outcome domain in each study were sought (e.g. for all measures, time points, analyses), and if not, the methods used to decide which results to collect. | Table 3 and 5 |
|  | 10b | List and define all other variables for which data were sought (e.g. participant and intervention characteristics, funding sources). Describe any assumptions made about any missing or unclear information. | Table 3 |
| Study risk of bias assessment | 11 | Specify the methods used to assess risk of bias in the included studies, including details of the tool(s) used, how many reviewers assessed each study and whether they worked independently, and if applicable, details of automation tools used in the process. | Page 2 section **Methods** Subsection **Risk of bias assessment** |
| Effect measures | 12 | Specify for each outcome the effect measure(s) (e.g. risk ratio, mean difference) used in the synthesis or presentation of results. | Table 5 |
| Synthesis methods | 13a | Describe the processes used to decide which studies were eligible for each synthesis (e.g. tabulating the study intervention characteristics and comparing against the planned groups for each synthesis (item #5)). | Page 2 section **Methods** Subsection **Study selection and data extraction** |
|  | 13b | Describe any methods required to prepare the data for presentation or synthesis, such as handling of missing summary statistics, or data conversions. | Page 2 section **Methods** Subsection **Data analysis and synthesis** |
|  | 13c | Describe any methods used to tabulate or visually display results of individual studies and syntheses. | Page 2 section **Methods** Subsection **Data analysis and synthesis** |
|  | 13d | Describe any methods used to synthesize results and provide a rationale for the choice(s). If meta-analysis was performed, describe the model(s), method(s) to identify the presence and extent of statistical heterogeneity, and software package(s) used. | Page 2 section **Methods** Subsection **Data analysis and synthesis** |
|  | 13e | Describe any methods used to explore possible causes of heterogeneity among study results (e.g. subgroup analysis, meta-regression). | We did not perform a meta-analysis |
|  | 13f | Describe any sensitivity analyses conducted to assess robustness of the synthesized results. | We did not perform a meta-analysis |
| Reporting bias assessment | 14 | Describe any methods used to assess risk of bias due to missing results in a synthesis (arising from reporting biases). | See Appendix 2 |
| Certainty assessment | 15 | Describe any methods used to assess certainty (or confidence) in the body of evidence for an outcome. | See Appendix 2 |
| **RESULTS** | | |  |
| Study selection | 16a | Describe the results of the search and selection process, from the number of records identified in the search to the number of studies included in the review, ideally using a flow diagram. | Figure 2 |
|  | 16b | Cite studies that might appear to meet the inclusion criteria, but which were excluded, and explain why they were excluded. | See below section Excluded studies with reasons |
| Study characteristics | 17 | Cite each included study and present its characteristics. | Table 3 |
| Risk of bias in studies | 18 | Present assessments of risk of bias for each included study. | Table 4 |
| Results of individual studies | 19 | For all outcomes, present, for each study: (a) summary statistics for each group (where appropriate) and (b) an effect estimate and its precision (e.g. confidence/credible interval), ideally using structured tables or plots. | Table 5 |
| Results of syntheses | 20a | For each synthesis, briefly summarise the characteristics and risk of bias among contributing studies. | Table 5 |
|  | 20b | Present results of all statistical syntheses conducted. If meta-analysis was done, present for each the summary estimate and its precision (e.g. confidence/credible interval) and measures of statistical heterogeneity. If comparing groups, describe the direction of the effect. | Table 5 |
|  | 20c | Present results of all investigations of possible causes of heterogeneity among study results. | We did not perform meta-analysis |
|  | 20d | Present results of all sensitivity analyses conducted to assess the robustness of the synthesized results. | We did not perform meta-analysis |
| Reporting biases | 21 | Present assessments of risk of bias due to missing results (arising from reporting biases) for each synthesis assessed. | See Appendix 2 for individual quality assessment |
| Certainty of evidence | 22 | Present assessments of certainty (or confidence) in the body of evidence for each outcome assessed. | See Appendix 2 for individual quality assessment |
| **DISCUSSION** | | |  |
| Discussion | 23a | Provide a general interpretation of the results in the context of other evidence. | Page 5 paragraphs 1, 2, and 3 |
|  | 23b | Discuss any limitations of the evidence included in the review. | See section Limitations on page 5 |
|  | 23c | Discuss any limitations of the review processes used. | See section Limitations on page 5 |
|  | 23d | Discuss implications of the results for practice, policy, and future research. | See section Conclusion on page 5 |
| **OTHER INFORMATION** | | |  |
| Registration and protocol | 24a | Provide registration information for the review, including register name and registration number, or state that the review was not registered. | Page 2, section **Methods**, subsection **Search strategy, inclusion and exclusion criteria** |
|  | 24b | Indicate where the review protocol can be accessed, or state that a protocol was not prepared. | Page 2, section **Methods**, subsection **Search strategy, inclusion and exclusion criteria** |
|  | 24c | Describe and explain any amendments to information provided at registration or in the protocol. | See below in section **Protocol deviations** |
| Support | 25 | Describe sources of financial or non-financial support for the review, and the role of the funders or sponsors in the review. | See section **Disclosure** on page 5 |
| Competing interests | 26 | Declare any competing interests of review authors. | See section **Disclosure** on page 5 |
| Availability of data, code and other materials | 27 | Report which of the following are publicly available and where they can be found: template data collection forms; data extracted from included studies; data used for all analyses; analytic code; any other materials used in the review. | No applicable |

## PRISMA 2020 for abstract checklist

| **Section and Topic** | **Item #** | **Checklist item** | **Reported (Yes/No)** |
| --- | --- | --- | --- |
| **TITLE** | | |  |
| Title | 1 | Identify the report as a systematic review. | Yes |
| **BACKGROUND** | | |  |
| Objectives | 2 | Provide an explicit statement of the main objective(s) or question(s) the review addresses. | Yes |
| **METHODS** | | |  |
| Eligibility criteria | 3 | Specify the inclusion and exclusion criteria for the review. | Yes |
| Information sources | 4 | Specify the information sources (e.g. databases, registers) used to identify studies and the date when each was last searched. | Yes |
| Risk of bias | 5 | Specify the methods used to assess risk of bias in the included studies. | Yes |
| Synthesis of results | 6 | Specify the methods used to present and synthesise results. | Yes |
| **RESULTS** | | |  |
| Included studies | 7 | Give the total number of included studies and participants and summarise relevant characteristics of studies. | Yes |
| Synthesis of results | 8 | Present results for main outcomes, preferably indicating the number of included studies and participants for each. If meta-analysis was done, report the summary estimate and confidence/credible interval. If comparing groups, indicate the direction of the effect (i.e. which group is favoured). | Yes |
| **DISCUSSION** | | |  |
| Limitations of evidence | 9 | Provide a brief summary of the limitations of the evidence included in the review (e.g. study risk of bias, inconsistency and imprecision). | Yes |
| Interpretation | 10 | Provide a general interpretation of the results and important implications. | Yes |
| **OTHER** | | |  |
| Funding | 11 | Specify the primary source of funding for the review. | No applicable |
| Registration | 12 | Provide the register name and registration number. | Yes |

## Protocol deviations

| Protocol section | Description | Deviation and justification |
| --- | --- | --- |
| Risk of bias quality assessment | Studies will be assessed using Cochrane's risk of bias tool for randomized control trials and ROBINS-I for non-randomized studies. | We did not use Cochrane's risk of bias tool for randomized control trials and ROBINS-I for non-randomized studies because we did not find those studies. We used the Quality In Prognosis Studies (QUIPS) tool, which includes questions that inform judgments of risk of bias in prognostic research. |
| Intervention(s), exposure(s). | We excluded generic instruments, clinician-reported, and patient-experience outcomes. | We included generic PROMs due the limited number of studies included. |
| Type and method of review | Meta-analysis | We were not able to perform meta-analysis due to the lack of data. |
| Setting | We will only include studies performed in the United States population. | We included studies not only from the United States due the limited number of studies included. |

## Search strategy and number of studies found

**Ovid Medline = 701**

1. patient-reported outcomes.mp. or exp Patient Reported Outcome Measures/

2. (patient reported outcome* or patient reported assessment* or patient reported symptom*).mp.

3. patient-reported outcomes.mp. or exp Patient Reported Outcome Measures/

4. (patient reported outcome* or patient reported assessment* or patient reported symptom*).mp.

5. (patient* adj1 (self-assess* or self-report* or self-monitor*)).mp.

6. (patient report* adj2 (outcome or measure or assessment or questionnaire or instrument or index or indice* or indicator*)).mp.

7. quality of life.mp. or "Quality of Life"/

8. Health related quality of life.mp.

9. (quality adj2 life).mp

10. (Health related adj3 quality adj3 life).mp.

11. 1 or 2 or 3 or 4 or 5 or 6 or 7 or 8 or 9 or 10

12. exp Heart Transplantation/ or heart transpl.mp.

13. (heart adj2 transplant*).mp.

14. (cardiac adj2 transplant*).mp.

15. ((heart or cardiac) adj3 transplant*).mp.

16. 12 or 13 or 14 or 15

17. 11 and 16

18. limit 17 to (english language and "all adult (19 plus years)")

**CINAHL Plus = 6,814**

(MH "Heart Transplantation+") OR "heart transplant" OR "cardiac transplant" AND (MH "Patient-Reported Outcomes+") OR "patient reported outcomes" OR (MH "quality of life") OR ("quality of life") OR ("health related quality of life")

**Web of Science = 1089**

#1 **TS=(patient-reported outcomes) OR TS=(patient-reported outcomes measure*) OR TS=(patient-reported assess*) OR TS=(patient-reported NEAR/3 (outcomes) ) OR TS=(patient-reported outcomes NEAR/4 (measure*) ) OR TS=(patient-reported NEAR/3 (assess*) ) OR TS=(patient-reported NEAR/3 (sympt*) ) OR TS=(patient-reported NEAR/3 (self*) ) OR TS=(patient-reported self*) OR TS=(patient-reported sympt*) OR TS=(quality of life) OR TS=(health related quality of life)**

#2 **TS = (heart AND transplant*) OR TS = (cardi* AND transplant*) OR TS=(heart NEAR/3 (transplant*) ) OR TS=(cardi* NEAR/3 (transplant*) )**

**#1 AND #2**

**Refined by: Document type: Articles; Countries/Regions: USA.**

**PubMed =5,377**

**(("patient reported outcome measures"[MeSH Terms]) OR (Patient Reported Outcomes[Text Word]) OR (quality of life[MeSH Terms]) OR (health related quality of life[Text Word])) AND (cardiac transplan*[Text Word]) OR (heart transplantation[Text Word]) OR ("heart transplantation"[MeSH Terms]) Filters: Case Reports, Clinical Study, Clinical Trial, Controlled Clinical Trial, Randomized Controlled Trial, Humans, English, Adult: 19+ years.**

**Last search: January 7, 2021.**

## Excluded studies with reasons

| Title | Year | Authors | Reason for exclusion |
| --- | --- | --- | --- |
| Qualitative interviews vs standardized self-report questionnaires in assessing quality of life in heart transplant recipients. | 2011 | Abbey, Susan E and De Luca, Enza and Mauthner, Oliver E and McKeever, Patricia and Shildrick, Margrit and Poole, Jennifer M and Gewarges, Mena and Ross, Heather J | Wrong outcome |
| Overall mental distress and health-related quality of life after solid-organ transplantation: results from a retrospective follow-up study. | 2013 | Baranyi, Andreas and Krauseneck, Till and RothenhÃ¤usler, Hans-Bernd | Wrong study design |
| Determinants of quality of life changes among long-term cardiac transplant survivors: Results from longitudinal data | 2003 | Barr, M L and Schenkel, F A and Van Kirk, A and Halbert, R J and Helderman, J H and Hricik, D E and Matas, A J and Pirsch, J D and Siegal, B R and Ferguson, R M and Nordyke, R J | Wrong study design |
| Measurement of health-related quality of life before and after heart-lung transplantation. | 1996 | Caine, N and Sharples, L D and Dennis, C and Higenbottam, T W and Wallwork, J | Wrong outcome |
| Impact of Fatigue Characteristics on Quality of Life in Patients After Heart Transplantation | 2017 | Chou, Yu-Ying and Lai, Yeur-Hur and Wang, Shoei-Shen and Shun, Shiow-Ching | Wrong study design |
| Comparison of Longterm Outcomes and Quality of Life in Recipients of Donation After Cardiac Death Liver Grafts With a Propensity-Matched Cohort | 2017 | Croome, K P and Lee, D D and Perry, D K and Burns, J M and Nguyen, J H and Keaveny, A P and Taner, C B | Wrong study design |
| Comparative analysis of the quality of life for patients prior to and after heart transplantation. | 2014 | Czyzewski, Lukasz and Torba, Krzysztof and Jasinska, Malgorzata and Religa, Grzegorz | Wrong study design |
| Perceived control and health-related quality of life in heart transplant recipients. | 2018 | Doering, Lynn V and Chen, Belinda and Deng, Mario and Mancini, Donna and Kobashigawa, Jon and Hickey, Kathleen | Wrong study design |
| Quality of life of advanced chronic heart failure: medical care, mechanical circulatory support and transplantation | 2016 | Emin, Akan and Rogers, Chris A and Banner, Nicholas R and Uk Cardiothoracic Transplant, Audit | Wrong outcome |
| Two-year follow-up of quality of life in patients referred for heart transplant | 2005 | Evangelista, L S and Dracup, K and Moser, D K and Westlake, C and Erickson, V and Hamilton, M A and Fonarow, G C | Wrong outcome |
| Functional status and perceived control influence quality of life in female heart transplant recipients | 2004 | Evangelista, L S and Moser, D and Dracup, K and Doering, L and Kobashigawa, J | Wrong study design |
| Changes in health-related quality-of-life and depression in heart-transplant recipients | 1995 | Fisher, D C and Lake, K D and Reutzel, T J and Emery, R W | Wrong outcome |
| Reliability and construct validity of PROMIS R measures for patients with heart failure who undergo heart transplant. | 2015 | Flynn, Kathryn E and Dew, Mary Amanda and Lin, Li and Fawzy, Maria and Graham, Felicia L and Hahn, Elizabeth A and Hays, Ron D and Kormos, Robert L and Liu, Honghu and McNulty, Mary and Weinfurt, Kevin P | Wrong study design |
| Improvement in quality of life in patients with heart failure who undergo transplantation | 1996 | Grady, K L and Jalowiec, A and WhiteWilliams, C | Wrong outcome |
| Predictors of quality of life in patients at one year after heart transplantation | 1999 | Grady, K L and Jalowiec, A and White-Williams, C | Wrong outcome |
| Quality of life 6 months after heart transplantation compared with indicators of illness severity before transplantation. | 1998 | Grady, K L and Jalowiec, A and White-Williams, C | Wrong study design |
| Predictors of quality-of-life in patients with advanced heart-failure awaiting transplantation | 1995 | Grady, K L and Jalowiec, A and Whitewilliams, C and Pifarre, R and Kirklin, J K and Bourge, R C and Costanzo, M R | Wrong study design |
| Patterns and predictors of quality of life at 5 to 10 years after heart transplantation | 2007 | Grady, K L and Naftel, D C and Kobashigawa, J and Chait, J and Young, J B and Pelegrin, D and Czerr, J and Heroux, A and Higgins, R and Rybarczyk, B and McLeod, M and White-Williams, C and Kirklin, J K | Wrong study design |
| The relationship between depressive symptoms and anxiety and quality of life and functional capacity in heart transplant patients. | 2007 | Karapolat, H and Eyigor, S and Durmaz, B and Yagdi, T and Nalbantgil, S and Karakula, S | Wrong study design |
| Health-related quality of life in long-term survivors after heart and lung transplantation: a prospective cohort study. | 2010 | Kugler, Christiane and Tegtbur, Uwe and Gottlieb, Jens and Bara, Christoph and Malehsa, Doris and Dierich, Martin and Simon, Andre and Haverich, Axel | Wrong outcome |
| Quality of life following transplantation of the heart, liver, and lungs | 1996 | Littlefield, C and Abbey, S and Fiducia, D and Cardella, C and Greig, P and Levy, G and Maurer, J and Winton, T | Wrong study design |
| Impact of symptom frequency and symptom distress on self-reported quality-of-life in heart-transplant recipients | 1987 | Lough, M E and Lindsey, A M and Shinn, J A and Stotts, N A | Lack full text |
| Life satisfaction following heart transplantation. | 1985 | Lough, M E and Lindsey, A M and Shinn, J A and Stotts, N A | Wrong study design |
| Getting old with a new heart: impact of age on depression and quality of life in long-term heart transplant recipients. | 2007 | Martinelli, Valentina and Fusar-Poli, Paolo and Emanuele, Enzo and Klersy, Catherine and Campana, Carlo and Barale, Francesco and Vigano, Mario and Politi, Pierluigi | Wrong study design |
| Health-related quality of life evolution in patients after heart transplantation. | 2008 | Martin-Rodriguez, A and Perez-San-Gregorio, M A and Diaz-Dominguez, R and Perez-Bernal, J | Wrong study design |
| Quality-of-life and coping in patients awaiting heart-transplantation | 1992 | Muirhead, J and Meyerowitz, B E and Leedham, B and Eastburn, T E and Merrill, W H and Frist, W H and Hoffman, F M and Augustine, S and Bergin, P | Wrong outcome |
| Quality of life of adults after a heart transplant. | 1989 | Packa, D R | Wrong study design |
| Health-related quality of life after different types of solid organ transplantation | 2000 | Pinson, C W and Feurer, I D and Payne, J L and Wise, P E and Shockley, S and Speroff, T | Wrong outcome |
| Lifestyle and quality of life in long-term cardiac transplant recipients | 2003 | Salyer, J and Flattery, M P and Joyner, P L and Elswick, R K | Wrong study design |
| Lifestyle and health status in long-term cardiac transplant recipients. | 2001 | Salyer, J and Sneed, G and MC, Corley | Wrong study design |
| Predicting quality of life with a pretransplantation assessment battery: A prospective study of cardiac recipients | 1995 | Sears, S F and Rodrigue, J R and Greene, A F and Mills, R M | Wrong study design |
| Health-related quality of life of transplant recipients: a comparison between lung, kidney, heart, and liver recipients | 2020 | Tarabeih, Mahdi and Bokek-Cohen, Ya'arit and Azuri, Pazit | Wrong study design |
